# Exploring the epidemiological burden of RSV pre- and post-COVID-19 pandemic: A Jordanian tertiary hospital experience

**DOI:** 10.1177/03000605241306405

**Published:** 2024-12-24

**Authors:** Enas Al-Zayadneh, Dana Marie, Farah A. Khraisat, Suzan S. Musa, Jehad Feras AlSamhori, Dina Alkhateeb Altamimi, Alia O. Khashman, Amirah Daher, Montaha AL-Iede

**Affiliations:** 1 Department of Pediatrics, Jordan University Hospital, Amman, Jordan; 2 School of Medicine, 54658 The University of Jordan, Amman, Jordan; 3 Department of Pediatrics, Alkhalidi Medical Center, Amman, Jordan; 4 Department of Pediatrics, The Ministry of Health, Amman, Jordan

**Keywords:** COVID-19, respiratory syncytial virus, lower respiratory tract infections, Jordan, epidemiology

## Abstract

**Objectives:**

To describe changes in respiratory syncytial virus (RSV) epidemiology, its associated clinical outcomes and predictors of severe acute lower respiratory tract infection (ALRTI) pre- and post-COVID-19.

**Methods:**

In this retrospective cohort, we analysed data from electronic medical record of children <5 years who were hospitalized at Jordan University Hospital with RSV-associated ALRTI from 2018 to 2022.

**Results:**

325 inpatients with respiratory infections were included. Rate of RSV infections decreased from 74% pre-pandemic to 30% post-pandemic. Patients diagnosed with ALRTI post-COVID had significantly higher SpO2, less chronic disease, lower temperature and respiratory rate at admission and fewer days in hospital compared with those diagnosed pre-COVID. Furthermore, patients diagnosed pre-pandemic were significantly more likely to have abnormal X-rays, used more antibiotics and antivirals, and had higher rates of severe disease than those with infection post-COVID.

**Conclusion:**

COVID-19 and its associated social restriction measures led to changes in RSV epidemiology, characterized by a decline in rates and clinical severity in the post-pandemic period. However, further studies are needed to characterize the impact of COVID-19 on subsequent RSV seasons.

## Introduction

Respiratory syncytial virus (RSV), a member of the pneumoviridae family, is a common global cause of acute lower respiratory tract infection (ALRTI).^1^ The virus affects both children and vulnerable groups including the elderly and those with weakened immune systems.^2,3^ Although RSV symptoms can vary from mild to severe, it places a considerable burden on children, being the leading cause of hospitalization in neonates and contributing to approximately 200,000 deaths annually among children under 5 years old in low-income countries.^4^ The World Health Organization (WHO) estimates that >60% of acute respiratory infections in children and >80% in infants younger than one year are due to RSV.^5^ In addition, RSV is linked to over 50% of hospitalized acute bronchiolitis cases in North America and nearly 40% of severe pneumonia cases in developing countries.^2^ Due to its burden, RSV infections are a pertinent public health issue. Importantly, the burden is believed to be partially associated with a lack of virus-specific vaccination.^6^

RSV is characterized by distinct seasonality, with the disease burden typically peaking during colder temperatures and higher humidity levels.^7^ In temperate countries, RSV is most common during the winter months, while in tropical and subtropical regions, it tends to peak during the rainy season.^3,8^ However, the outbreak of the COVID-19 pandemic significantly intervened with the seasonality of RSV and its burden by association. Studies indicate that in 2020, the typical autumn and winter RSV epidemics were nearly absent, with some countries experiencing an unprecedented shift in the RSV season to spring.^9^ Additionally, in countries with cold and dry climates, RSV has shown an unusual pattern, with a large early epidemic followed by a smaller, delayed epidemic the following year.^10^ Due to the similar route of transmission between COVID-19 and RSV, public and social health measures (e.g., masks, social distancing, hand hygiene) were believed to have an enormous impact on viral diseases during the pandemic.^11^ Furthermore, widespread changes in medication-related behaviours, such as the increased use of antibiotics, have also been linked to alterations in RSV seasonality.^12^

Interestingly, these shifts in RSV seasonality were linked to distinct clinical outcomes. For instance, delayed RSV outbreaks were linked to high hospitalization rates, even though the clinical presentations were mild.^6,7,9–11^ Therefore, in the post-pandemic era, it is crucial to understand the changes in RSV seasonality and their associated clinical outcomes, to optimize treatment strategies and anticipate control measures if similar shifts in seasonality occur in the future. This study aimed to characterize the epidemiology of RSV, its associated clinical outcomes, and the predictors of severe ALRTI, based on data extracted from a cohort of hospitalized paediatric patients at a tertiary hospital in Jordan.

## Methodology

### Patients

This retrospective, observational study was conducted among hospitalised patients at Jordan University Hospital, a tertiary care facility located in Amman, Jordan. The study covered the periods from 1 January 2018 to 11 March 2020 (pre COVID) and from 11 March 2020 to 31 December 2022 (post-COVID).

Children under 5 years of age who had undergone nasopharyngeal aspiration (NPA) followed by viral polymerase chain reaction (PCR) testing, and, had tested positive for RSV were included in the study. Nasopharyngeal swabs were collected in Copan universal transport and preservation media and stored at −20°C. Conventional real-time PCR (FTD Respiratory Pathogens 21 Assay Kit and EZ 1 and 2 Virus Mini Kit V2.0 by Qiagen), was used for the nucleic acid extraction. Viruses detected included: RSV; adenovirus (AdV-B, AdV-C); rhinovirus (A, B &C); enterovirus; parainfluenza viruses 1–4; rotavirus; parechovirus; bocavirus; metapneumovirus; influenza (type A, B, C, and A/B); Coronavirus SARS-CoV-2.

The reporting of this study conforms to STROBE guidelines.^13^ The study obtained formal approval from the University of Jordan Institutional Review Board (Approval #2022-2023/6). Written informed consent was not required due to the retrospective design of the study and patient data were anonymized prior to analysis.

### Clinical data

The following data were extracted from the patients’ electronic medical records: age; sex; prematurity (defined as gestational age <37 weeks); low birth weight (defined as birth weight <2.5 kg);^14^ neonatal intensive care unit (NICU) admission; infection exposure; smoking exposure; presence of chronic disease; received flu vaccine; antibiotic use before hospitalization.

Other data (collected on admission) included: oxygen saturation (SpO^2^); temperature; respiratory rate (RR); presence of signs of respiratory distress (e.g., grunting, nasal flaring, retractions); results of plain radiographs (reviewed and interpreted by a radiologist blinded to patients’ clinical data). In addition, the following were noted: admission to paediatric intensive care unit (PICU); length of hospital stay (LoS); mechanical ventilation requirement; use of oxygen supplementation; duration of oxygen supplementation; antibiotic use; corticosteroid use; antiviral drug use.

Patients were classified as having severe ALRTI if they had a cough or difficulty in breathing in addition to one or more of the following: grunting; nasal flaring; retractions; SpO^2^ <90%; ICU admission; severe tachypnoea (i.e., respiratory rate >60/min); death.^15^

### Statistical analysis

Statistical analysis was performed using SPSS software (version 26.0 for Windows®; IBM Corp, Armonk, NY, USA). A *P*-value <0.5 was considered to indicate statistical significance. Data were described in terms of median and range, or absolute numbers and percentages as appropriate. Comparison of categorical variables between groups were performed using χ^2^ tests.

Comparison of continuous variables between groups was performed using Mann Whitney *U* test for independent samples or Kruskal-Wallis test, as appropriate. Predictors of severe ALRTI were assessed using a multivariate binary logistic regression model. Results were presented as odds ratio (OR) and 95% confidence intervals (CIs).

## Results

**Figure 1.**
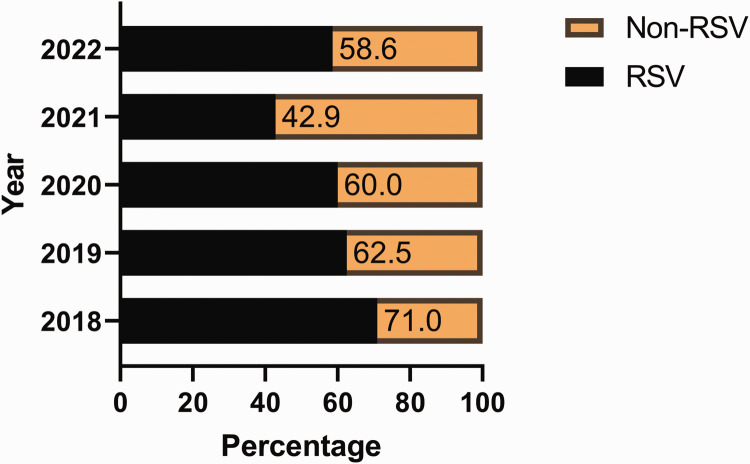
Distribution of in-patient respiratory infections from 2018 to 2022.

**Figure 2.**
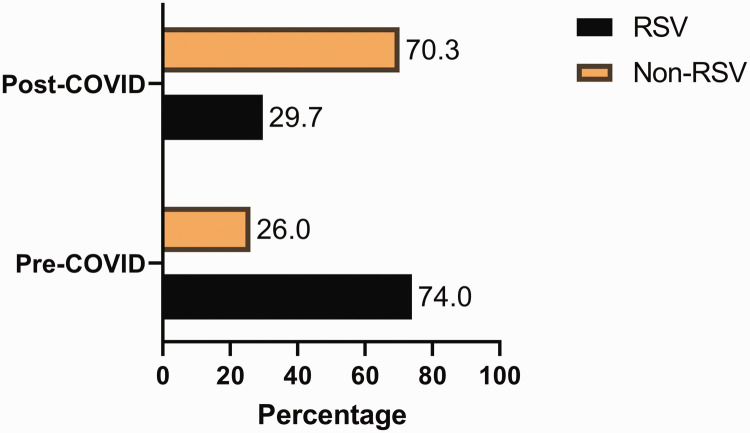
Distribution of in-patient respiratory pre- and post-COVID pandemic.

In total, 325 hospitalised paediatric patients with ALRTI were included. The incidence of RSV infections decreased from 71% in 2018 to 43% in 2021, but increased to 59% in 2022. (Figure 1). The rate of RSV infections decreased from 74% pre COVID to 30% post COVID (Figure 2). By contrast, non-RSV infections increased from 26% pre COVID to 70% post COVID.

Although age and sex ratio of the patients did not differ across the years, other clinical characteristics were significantly different (Table 1). For example, there were significant differences among, prematurity, low birth rate, breast feeding, infection exposure, smoking exposure, and incidence of chronic disease. Other clinical variables with significant differences included: SpO^2^; temperature at diagnosis; RR at diagnosis; LoS; respiratory distress; abnormal x-ray findings; PICU/NICU admissions; O^2^ supplementation; duration of O^2^ supplementation. The rate of medications (i.e., antibiotics, corticosteroids, antiviral drugs) was also significantly different across the years. Finally, the incidence of severe ALRTI significantly differed across the years.

**Table 1.** Characteristics of participants by year of admission.

Characteristics	Total(N = 325)	2018(N = 45)	2019(N = 58)	2020(N = 80)	2021(N = 47)	2022(N = 95)	Statistical significance
Age, months	5.0 (2.0–10.0)	3.5 (1.5–8.0)	5 (2.5–12.0)	4 (1.6–9.0)	6 (3.0–12.0)	5 (1.5–11.0)	ns
Sex, M: F	191/134	23/22	38/20	47/33	31/16	52/43	ns
Prematurity	62/301 (21)	11 (24)	16 (36)	13 (17)	5 (11)	17 (19)	*P* = 0.047
Low birth weight	69/291 (24)	10 (22)	19 (41)	12 (17)	8 (17)	20 (24)	*P* = 0.033
NICU admission	102/313 (33)	12 (27)	19 (39)	25 (33)	11 (23)	35 (37)	ns
Breastfeeding	39/144 (27)	12 (100)	2 (100)	4 (10)	11 (32)	10 (19)	*P < *0.000
Daycare attendance	19/176 (11)	0 (0)	1 (10)	7 (11)	3 (8)	8 (16)	ns
Infection exposure	99/186 (53)	2 (17)	3 (27)	37 (54)	24 (67)	33 (57)	*P* = 0.015
Smoking exposure	40/122 (33)	0 (0)	1 (14)	13 (33)	10 (29)	16 (50)	*P* = 0.048
Chronic disease	80/325 (25)	14 (31)	23 (40)	17 (21)	3 (6)	23 (24)	*P* = 0.002
Flu vaccine	3/265 (1)	0 (0)	0 (0)	2 (3)	0 (0)	1 (1)	NA
Antibiotics (previous)	37/266 (14)	1 (5)	6 (13)	15 (20)	2 (5)	13 (16)	ns
SpO2	91.2 (88.0–95.0)	86.5 (80.0–89.5)	90 (86.0–94.0)	91 (88.0–96.0)	95 (93.0–97.0)	93 (89.0–96.0)	*P < *0.000
Temperature at admission, °C	37.0 (36.6–38.0)	38.5 (38.0–38.5)	38.7 (38.5–39.2)	37 (36.7–37.1)	37.7 (36.6–38.0)	36.8 (36.6–37.6)	*P < *0.000
RR at admission, breaths/min	50.0 (40.0–60.0)	50 (47.0–65.0)	53 (40.0–65.0)	50 (40.0–60.0)	50 (44.0–50.0)	45 (33.5–55.0)	*P* = 0.011
Length of stay, days	6 (4–9)	7 (5–9)	6 (4–8)	7 (5–9)	4 (3–5)	6 (4–10)	*P < *0.000
Respiratory distress	204/323 (63)	42 (93)	34 (59)	40 (50)	22 (47)	66 (71)	*P < *0.000
Abnormal X-ray	211/287 (74)	38 (95)	42 (81)	45 (60)	27 (61)	59 (78)	*P* = 0.000
X-ray findings							NA
*Missing*	38/325 (12)	5 (11)	6 (10)	5 (6)	3 (6)	19 (20)	ns
*Consolidation*	43/325 (13)	7 (16)	9 (16)	14 (18)	0 (0)	13 (14)	ns
*Hyperinflation*	62/325 (19)	10 (22)	11 (19)	25 (31)	11 (23)	5 (5)	ns
*Interstitial infiltrates*	106/325 (33)	21 (47)	22 (38)	6 (8)	16 (34)	41 (43)	ns
*Normal*	76/325 (23)	2 (4)	10 (17)	30 (38)	17 (36)	17 (18)	ns
PICU/NICU admission	156/322 (48)	27 (61)	18 (31)	55 (71)	6 (13)	50 (53)	*P < *0.000
Mechanical ventilation	23/323 (7)	5 (11)	3 (5)	7 (9)	0 (0)	8 (8)	ns
O2 supplementation	240/273 (88)	44 (98)	39 (77)	77 (99)	40 (87)	40 (76)	*P < *0.000
Duration O2 supplementation, days	4 (2–7)	5 (3–7)	5 (2–8)	5 (3–7)	3 (2–4)	4 (2–7)	*P < *0.000
Antibiotics	251/315 (80)	41 (93)	53 (91)	63 (81)	28 (68)	66 (70)	*P* = 0.001
Corticosteroids	186/315 (59)	16 (36)	38 (66)	54 (69)	29 (71)	49 (52)	*P* = 0.001
Antiviral drugs	67/314 (21)	16 (36)	13 (22)	26 (33)	6 (15)	6 (7)	*P < *0.000
severe ALRTI	244/322 (76)	43 (96)	44 (77)	62 (78)	23 (51)	72 (76)	*P < *0.000

Data are expressed as n/N (%) or median (interquartile range).

NICU, neonatal intensive care unit; PICU, paediatric intensive care unit; SpO2, oxygen saturation; RR, respiratory rate; ALRTI, acute lower respiratory tract infection; not statistically significant

Comparison of clinical variables over the years separated into severe and non-severe ALRTI groups, showed significant differences between the groups (Table 2). For example, by comparison with patients with non-severe disease, patients with severe disease were more likely to be younger (*P* < 0.001), premature (*P* < 0.001), have low birth weight (*P* = 0.007), and a history of NICU admissions (*P* = 0.001). In terms of clinical characteristics, the non-severe disease group had significantly higher SpO^2^, lower RR, lower LoS, fewer cases of respiratory distress, and fewer abnormal X-ray findings (all *P* < 0.001) than the severe disease group. Importantly, patients in the severe disease group had significantly more PICU/NICU admissions (*P* < 0.001), requirement for mechanical ventilation (*P* = 0.02), O^2^ supplementation (*P* < 0.001), and greater duration of O^2^ supplementation (*P* < 0.001) than patients in the non-severe disease group. In terms of medications, by comparison with patients in the non-severe group, patients with severe disease had more antibiotics (*P* = 0.028) and antiviral drugs (*P* = 0.012).

**Table 2.** Characteristics of participants stratified by clinical status (N = 322*).

Characteristics	Non severe ALRTI(N = 78)	Severe ALRTI(N = 244)	Statistical significance
Age, months	7 (4.0–15.0)	4 (1.6–9.0)	*P < *0.000
Sex, M: F	48/30	141/103	ns
Prematurity	3 (4)	58 (25)	*P < *0.000
Low birth weight	7 (11)	62 (28)	*P* = 0.007
NICU admission	13 (18)	89 (38)	*P* = 0.001
Breastfeeding	9 (24)	30 (29)	ns
Daycare attendance	6 (15)	13 (10)	ns
Infection exposure	30 (63)	68 (50)	ns
Smoking exposure	11 (28)	28 (35)	ns
Chronic disease	21 (27)	59 (24)	ns
Flu vaccine	0 (0)	3 (2)	NA
Antibiotics (previous)	10 (15)	26 (13)	ns
SpO2	95 (94.0–97.0)	90 (85.0–95.0)	*P < *0.000
Temperature at admission, °C	37 (36.7–38.0)	37 (36.6–38.0)	ns
RR at admission, breaths/min	40 (32.0–50.0)	50 (44.0–62.0)	*P < *0.000
Length of stay, days	4 (3.0–5.0)	7 (5.0–10.0)	*P < *0.000
Respiratory distress	1 (1)	203 (83)	*P < *0.000
Abnormal X-ray	35 (52)	176 (81)	*P < *0.000
X-ray findings			NA
*Missing*	10 (13)	27 (11)	
*Consolidation*	4 (5)	39 (16)	
*Hyperinflation*	13 (17)	49 (20)	
*Interstitial infiltrates*	18 (23)	88 (36)	
*Normal*	33 (42)	41 (17%)	
PICU/NICU admission	9 (12)	147 (61)	*P < *0.000
Mechanical ventilation	1 (1)	22 (9)	*P* = 0.020
O2 supplementation	45 (69)	194 (95)	*P < *0.000
Duration O2 supplementation, days	2 (1.0–4.0)	5 (3.0–7.0)	*P < *0.000
Antibiotics	52 (71)	199 (83)	*P* = 0.028
Corticosteroids	45 (62)	140 (58)	ns
Antiviral drugs	8 (11)	59 (25)	*P* = 0.012

Data are expressed as n/N (%) or median (interquartile range).

* Data missing for 3 patients

NICU, neonatal intensive care unit; PICU, paediatric intensive care unit; SpO2, oxygen saturation; RR, respiratory rate; ALRTI, acute lower respiratory tract infection; not statistically significant

Interestingly, the rate of co-infection with bacteria or other viruses did not significantly differ between groups (non-severe, 28%, severe 29%). Co-infection with rhinovirus was most frequently observed in both groups (non-severe, 14%, severe 11%). Next most common was adenovirus (non-severe, 4%, severe 2%) followed by coronavirus SARS-CoV-2 (non-severe, 1%, severe 4%).

Compared with patients diagnosed pre-COVID, fewer patients diagnosed post-COVID were premature or breastfed (*P* < 0.05). Moreover, patients diagnosed post-COVID had significantly higher SpO2, less chronic disease, lower temperature and RR at admission and LoS compared with those diagnosed pre-COVID (all *P* < 0.05; Table 3). Furthermore, patients diagnosed post-COVID were significantly less likely to have abnormal X-rays (*P* < 0.001), PICU/NICU admission (*P* = 0.016), require O^2^ supplementation long-term (*P* < 0.001), require antibiotics (*P* < 0.001), or antiviral drugs (*P* < 0.001) compared with those diagnosed pre-COVID. Interestingly, patients diagnosed post-COVID had reduced rates of severe ALRTI (*P* = 0.033).

**Table 3.** Characteristics of participants stratified according to pandemic status.

Characteristics	Pre-COVID(n = 148)	Post-COVID(n = 177)	Statistical significance
Age, months	5 (2.0–10.0)	5 (1.7–10.0)	ns
Sex, M: F	92/56	99/78	ns
Prematurity	34 (26)	28 (17)	*P* = 0.044
Low birth weight	37 (28)	32 (20)	ns
NICU admission	44 (32)	58 (33)	ns
Breastfeeding	16 (47)	23 (21)	*P* = 0.003
Daycare attendance	8 (13)	11 (10)	ns
Infection exposure	27 (44)	72 (58)	ns
Smoking exposure	10 (30)	30 (34)	ns
Chronic disease	51 (35)	29 (16)	*P < *0.000
Flu vaccine	2 (2)	1 (1)	ns
Antibiotics (previous)	12 (11)	25 (16)	ns
SpO2	88.5 (85.0–92.0)	95 (90.0–96.0)	*P < *0.000
Temperature at admission, °C	37.5 (37.0–38.7)	36.9 (36.6–37.9)	*P < *0.000
RR at admission, breaths/min	52 (45.0–63.0)	47 (37.0–55.5)	*P < *0.000
Length of stay, days	7 (4.0–9.0)	5 (4.0–8.0)	*P* = 0.017
Respiratory distress	97 (66)	107 (61)	ns
Abnormal X-ray	112 (84)	99 (65)	*P < *0.000
X-ray findings			NA
*Missing*	14 (10)	24 (14)	
*Consolidation*	26 (18)	17 (10)	
*Hyperinflation*	42 (28)	20 (11)	
*Interstitial infiltrates*	44 (30)	62 (35)	
*Normal*	22 (15)	54 (31)	
PICU/NICU admission	81 (56)	75 (42)	*P* = 0.016
Mechanical ventilation	13 (9)	10 (6)	ns
O2 supplementation	126 (90)	114 (86)	ns
Duration O2 supplementation, days	5 (3.0–7.0)	4 (2.0 –5.0)	*P* = 0.002
Antibiotics	130 (88)	121 (72)	*P < *0.000
Corticosteroids	91 (62)	95 (57)	ns
Antiviral drugs	46 (31)	21 (13)	*P < *0.000
severe ALRTI	51 (35)	42 (24)	*P* = 0.033

Data are expressed as n/N (%) or median (interquartile range).

NICU, neonatal intensive care unit; PICU, paediatric intensive care unit; SpO2, oxygen saturation; RR, respiratory rate; ALRTI, acute lower respiratory tract infection; not statistically significant

Multivariate analysis showed that febrile status at admission (OR: 2.92; 95%CI: 1.01–8.41), abnormal X-ray findings (OR: 3.63; 95% CI: 1.49–8.91), PICU/NICU admissions (OR: 4.31; 95% CI: 1.46–12.80), O^2^ supplementation (OR: 5.10; 95% CI: 1.30–19.93), and increased length of hospitalization (OR: 1.21; 95% CI: 1.00–1.46) were significantly positive predictors of severe ALRTI (*P* < 0.05; Table 4).

**Table 4.** Predictors of severe ALRTI.

Variable	Odds Ratio	95% CIs	Statistical significance
Prematurity	3.21	0.48–21.7	ns
Low birth weight	0.94	0.25–3.49	ns
Febrile at admission	2.92	1.02–8.41	*P* = 0.047
Abnormal X-ray findings	3.64	1.49–8.91	*P* = 0.005
PICU/NICU admission	4.32	1.46–12.80	*P* = 0.008
O^2^ supplementation	5.10	1.30–19.93	*P* = 0.019
Antibiotic use	1.16	0.42–3.20	ns
Corticosteroid use	0.97	0.41–2.28	ns
Antiviral drug use	1.24	0.39–3.99	ns
Length of hospital stay	1.21	1.00–1.46	*P* = 0.050

ALRTI, acute lower respiratory tract infection; NICU, neonatal intensive care unit; PICU, paediatric intensive care unit; CIs, confidence intervals; not statistically significant

## Discussion

The results of this study indicate that the incidence of ALRTI fluctuated throughout the data collection period. It decreased during the COVID-19 pandemic years, with a gradual return to pre-pandemic levels beginning in 2022. Patients with severe ALRTI were younger, premature at birth, had lower birth weight, and required more interventions than patients with non-severe ALRTI. However, co-infection rates did not differ by disease severity. Multivariate analysis showed predictors of severe disease included febrile status and abnormal X-rays at admission, PICU/NICU stay, and oxygen supplementation. Interestingly, patients diagnosed pre-pandemic with severe ALRTI, experienced more severe disease, longer hospitalization, and higher antibiotic and antiviral use compared with those diagnosed post-pandemic.

The shift in the epidemiology and seasonality of RSV during the COVID-19 pandemic has been reported elsewhere.^1,3,16–19^ Indeed, analysis of the Virus Detection Surveillance System data showed that weekly RSV cases in the U.S. and Canada decreased by nearly 100-fold.^3^ Furthermore, several South American and European nations reported no cases of RSV during the COVID-19 pandemic.^3^ This phenomenon was linked to several factors, primarily the introduction of stringent public health measures, intra-viral competition and viral interference, as well as pandemic-related disruptions in healthcare logistics.^1,4,12^ Social restrictions, such as physical distancing, school closures, and limits on public interactions, were identified as primary factors driving changes in RSV seasonality. Complemented by increased hygiene standards during the pandemic, these social restrictions contributed to the reduction of RSV transmission through droplets or contact.^12^ However, the decrease in RSV cases may have been due to the purely logistical issue of a globally over-burdened healthcare system. For example, during the pandemic, patients with mild-to-moderate disease were less likely to attend face-to-face services, would only visit emergency departments for life-threatening cases and, tended to rely on tele-consultations.^4^ While reduced testing could have been a potential explanation for the decline in RSV cases, this was not the case, as the number of RSV tests actually increased during that period.^20,21^

Intra-viral competition and its impact on RSV cases has previously been demonstrated in a number of non-COVID instances. For example, during the 2009 influenza pandemic, the Hong Kong H1N1 outbreak, and the adenovirus epidemic in Hunan, delayed peaks in RSV activity were observed in comparison.^22–24^ Significantly, viral interactions, particularly those involving rhinovirus, were thought to have played a role in delaying the onset of COVID-19 in Europe.^25^ Therefore, it is conceivable that once pandemic-related viral interactions stabilized, they may have influenced RSV epidemics.

Consistent with global trends, we have observed a notable decline in severe RSV cases in Jordan during the years following the pandemic. This particular phenomenon is poorly understood and has been attributed to a number of different factors. For instance, some studies have linked the reduction in severity to the older age of infected individuals, as older patients tend to have more resilient airways and stronger immune responses than younger patients.^26^ This aligns with the typical age-related patterns of RSV, which tends to cause mild illness in older individuals but can result in more severe or life-threatening outcomes in young children, particularly those with weakened immune systems.^27^ Additionally, it has been suggested that variations in RSV severity could be linked to reduced viral activity or geographic factors that may impact the spread and development of different RSV strains.^11^ Finally, one study suggested that this shift could be due to the new hygiene practices adopted by parents during the pandemic.^12^

Interestingly, we observed an increase in non-RSV cases in the post-pandemic years. Therefore, our findings are consistent with studies that indicated that the flu virus was more effectively suppressed during the COVID period than RSV.^4^ This observation implies that the transmission dynamics and viral burden, in the context of implemented public health measures and viral interactions, may differ substantially from one virus to another. However, co-infection is a complex phenomenon, with reported rates varying widely, from nearly absent in some studies to significantly high in others.^3^ Additionally, it has been observed that pre-pandemic RSV infections were often complicated by secondary bacterial infections, especially in young patients.^28^ Notably, we did not observe a statistically significant effect of co-infections on disease severity of ALRTI.

The pattern of RSV resurgence in several countries during the summer of 2021 has been documented in other studies.^1,3^ In contrast to historical trends, these atypical RSV resurgences began earlier in the year and lasted longer than previously noted. This phenomenon has been attributed to a number of factors. For example, the relaxation and inconsistent enforcement of social restrictions played a key role. This factor also underscored the potential contribution of adults in the transmission of RSV.^29^ It was observed that the increase in RSV cases coincided more with the easing of social restrictions and the reopening of borders than with the reopening of schools and childcare facilities. Additionally, the low level of RSV activity left previously unaffected children vulnerable to an ‘immunity debt’, which impaired their ability to handle severe disease thereafter.^30,31^ This also resulted in decreased adult immunity to RSV. Another factor could be the changes in climate and the age distribution of the participants, which were believed to have impacted both the transmission and severity of RSV.^32,33^ It is important to highlight that in our study, the median age at RSV diagnosis did not show a significant difference between the pre- and post-pandemic periods. Overall, the current literature does not link this resurgence to the emergence of a new RSV strain.^3,34^

In the context of Jordan, the kingdom implemented early and highly stringent lockdown measures. In early March 2020, the Jordanian government imposed a full national lockdown, which was later relaxed to a curfew allowing only designated family members to travel for essential purchases.^35^ Additionally, the use of cars was restricted, with exceptions made only for healthcare workers and those in essential sectors. Jordan also imposed fines ranging from $30 to $70 for individuals who failed to adhere to social distancing measures, particularly the requirement to wear masks in public spaces. While these initial measures helped control the COVID-19 infection rate, the relaxation of restrictions in the subsequent months, coupled with the reopening of the economy, contributed to a surge in COVID-19 cases and increased associated case-fatality rates.^36^ During the pandemic period, both private and public hospitals were primarily focused on treating COVID-19 cases or high-risk clinical cases with COVID-19-related complications. In addition, technical resources were redirected towards monitoring and analysing COVID-19 data within the kingdom. We are of the opinion that while the efforts to minimize the risk of COVID-19 were commendable, their shortcomings ultimately proved detrimental not only to the economy but also to the entire Jordanian healthcare system. The impact led to a shift in the responsibility for managing common illnesses, such as respiratory infections, from healthcare institutions to individuals. As a result, the burden of these diseases could either have been reduced due to decreased transmission pathways associated with strict preventive measures, or it may have been obscured due to insufficient follow-up and the overwhelming strain placed on the healthcare system by COVID-19.

Our study had several limitations, one of which was the absence of data from the last two seasons, covering the period from December 2022 to 2024. Other notable limitations of our study included a modest sample size, data from a single centre, its retrospective design, and reliance on data from electronic medical records. Moreover, the study lacked RSV typing, and we were unable to demonstrate viral or bacterial distribution among the participants. We were also unable to distinguish between superinfections and early co-infections. Further studies involving large numbers of patients and control groups are required to confirm our results.

In conclusion, consistent with global findings, the COVID-19 pandemic and the socio-behavioural changes it brought about had a significant impact on the epidemiology and clinical manifestations of RSV at a tertiary hospital in Jordan. Further research is needed to uncover the molecular mechanisms behind inter- and intra-virus interactions, as their consequences influence clinical, diagnostic, and therapeutic management decisions. Nevertheless, given the dynamic nature of viral infections, including variations in transmission, shedding duration, host immune responses, and socio-environmental factors, modelling the interactions between viruses is extremely challenging. It is anticipated that populations living in different climates will experience distinct epidemic dynamics, particularly in terms of disease progression and severity.
